# The mechanisms of regulatory T cells in the immune microenvironment of multiple myeloma and clinical significance

**DOI:** 10.3389/fimmu.2026.1830089

**Published:** 2026-04-29

**Authors:** Lijun Du, Yumeng Jiang, Qiaolin Zhou, Fang Xu

**Affiliations:** 1 Department of Hematology, Mianyang Central Hospital, School of Medicine, University of Electronic Science and Technology of China, Mianyang, China; 2 School of Medicine, University of Electronic Science and Technology of China, Chengdu, China

**Keywords:** immunotherapy, multiple myeloma, pathogenesis, regulatory T cells, tumor immune microenvironment

## Abstract

Multiple Myeloma is a malignant hematologic disorder characterized by the abnormal proliferation of clonal plasma cells. Its pathogenesis involves a variety of complex factors, with particular attention given to the immune regulatory mechanisms within the tumor microenvironment (TME). Research indicates that the immunosuppressive microenvironment provides a protective barrier for the survival and progression of MM cells. In this context, regulatory T cells (Tregs), as key immunosuppressive cells within the TME, play a crucial role in the tumor immune tolerance network. Through interactions with various immune cells and tumor-associated factors, Tregs participate in the regulation of MM-related signaling pathways and mediate tumor angiogenesis, thereby promoting immune evasion of MM cells. This review aims to explore the specific roles and clinical significance of Tregs in the immune mechanisms of MM.

## 1 Introduction

Multiple myeloma (MM) is a malignant proliferative disease of plasma cells, characterized by the clonal expansion of plasma cells, leading to clinical manifestations such as anemia, bone destruction, hypercalcemia, and renal failure. MM accounts for approximately 10%-15% of hematologic malignancies, making it the second most common malignancy of the hematologic system, following non-Hodgkin lymphoma (1, 2). Despite the significant improvement in treatment response rates for MM achieved through hematopoietic stem cell transplantation (HSCT) and targeted therapies such as proteasome inhibitors (PIs), immunomodulatory drugs (IMiDs), and monoclonal antibodies (mAbs), the disease remains inherently incurable as a malignant neoplasm. The vast majority of patients ultimately experience relapse or develop drug resistance (3, 4). The mechanisms underlying relapse and drug resistance in MM primarily involve two aspects: the intrinsic molecular regulatory mechanisms within tumor cells and the regulatory role of the tumor microenvironment (TME). The TME contributes to disease progression through several key mechanisms: intercellular interactions within the bone marrow microenvironment, hypoxia-induced promotion of tumor cell survival and drug resistance, and the upregulation of immunosuppressive cells and immune checkpoint molecules that inhibit anti-tumor immune responses, thereby leading to immune escape of tumor cells (5–7). The TME of MM consists of effector cells, such as conventional T cells, natural killer (NK) cells, γδ T cells, and NKT cells; immunosuppressive cells, including regulatory T cells (Tregs), regulatory B cells (Bregs), and myeloid-derived suppressor cells (MDSCs); and cells that acquire protumoral functions under the influence of the TME, such as bone marrow stromal cells (BMSCs), endothelial cells, osteoblasts (OBs), and osteoclasts (OCs) (8). The TME not only provides essential survival support for tumor cells but also mediates the development of drug resistance through complex intercellular interactions, offering a crucial theoretical foundation and research direction for the development of novel targeted therapeutic strategies.

Tregs are a distinct subset of CD4+ T lymphocytes, primarily involved in maintaining self-tolerance and immune homeostasis, and are characterized by the intracellular expression of the forkhead box protein P3 (Foxp3) transcription factor (9). Additionally, Tregs can be exploited by tumor cells to mediate immune escape, making them one of the key components of the tumor immune tolerance network within the immune microenvironment of MM.

The highly immunosuppressive nature of the MM bone marrow microenvironment has garnered significant attention over the past decade, with increasing focus on the pivotal role of Tregs in this suppression. Tregs promote their own proliferation and activation through various mechanisms, including cell-to-cell contact, such as cytotoxic T lymphocyte-associated protein 4 (CTLA-4)/B7, programmed cell death protein 1 (PD-1)/programmed death-ligand 1 (PD-L1) interactions, and the secretion of immunosuppressive cytokines, such as interleukin-10 (IL-10) and transforming growth factor β (TGF-β), thereby inhibiting the function of effector T cells (Teffs) and NK cells (10). Additionally, Tregs interact with other immunosuppressive cells, such as MDSCs and tumor-associated macrophages (TAMs), further remodeling the tumor immune microenvironment (11). These mechanisms collectively enable MM cells to evade immune surveillance, promoting their survival, proliferation, metastasis, and drug resistance, ultimately driving disease progression.

**Figure 1:**
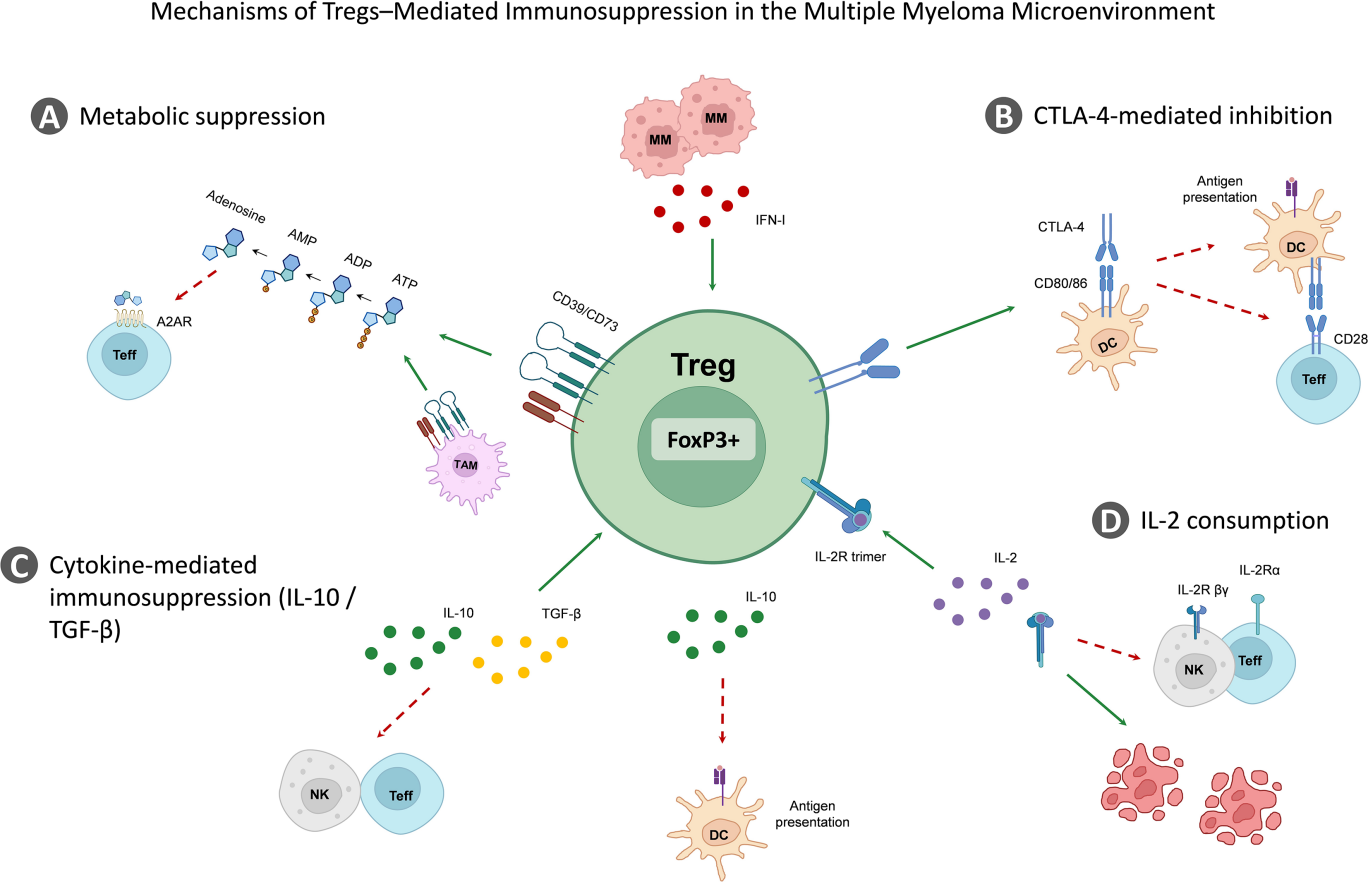
Mechanisms of regulatory T cell–mediated immunosuppression in the multiple myeloma microenvironment. **(A)** Metabolic suppression: Tregs express the ectonucleotidases CD39 and CD73, which convert extracellular ATP and ADP into AMP and subsequently into adenosine. Adenosine engages A2A receptors on Teffs, leading to inhibition of T-cell activation and function. **(B)** CTLA-4–mediated inhibition: Tregs express high levels of CTLA-4, which binds CD80/CD86 on dendritic cells (DCs), thereby limiting CD28-mediated co-stimulation and impairing antigen presentation to effector T cells. **(C)** Cytokine-mediated immunosuppression: Tregs secrete immunosuppressive cytokines such as IL-10 and TGF-β, which suppress the activity of NK cells and effector T cells, inhibit dendritic cell function, and promote Treg expansion. **(D)** IL-2 consumption: Tregs express the high-affinity IL-2 receptor (IL-2Rα/β/γ), allowing them to efficiently consume IL-2 in the tumor microenvironment and limit its availability for effector immune cells, thereby weakening antitumor immunity. Treg, regulatory T cell; Teffs, effector T cells; NK, natural killer cell; DC, dendritic cell; CTLA-4, cytotoxic T-lymphocyte–associated antigen 4; IL-2, interleukin-2; TGF-β, transforming growth factor-β; A2AR, adenosine A2A receptor.

This article reviews the mechanisms of Tregs in the immune microenvironment of MM, as illustrated in **Figure 1**, **Supplementary Figure S1** clinical significance.

## 2 Mechanisms of treg-mediated immunosuppression in MM

It should be noted that many immunosuppressive mechanisms attributed to regulatory T cells (Tregs) have been extensively characterized in general immunology and across diverse tumor types. These include the secretion of inhibitory cytokines (e.g., IL-10 and TGF-β), metabolic disruption via the CD39/CD73–adenosine axis, and CTLA-4–mediated inhibition of antigen-presenting cells. However, despite being well established as canonical pathways of Treg-mediated immune suppression, direct evidence supporting their specific functional roles within the multiple myeloma (MM) bone marrow microenvironment remains incomplete. In several cases, current understanding relies on extrapolation from other malignancies or experimental systems. Therefore, to improve conceptual clarity and rigor, we distinguish throughout this review between mechanisms that have been directly validated in MM and those that remain inferred based on broader Treg biology, according to the strength of available evidence (**Table 1**).

**Table 1:** Classification of Treg-mediated immunosuppressive mechanisms in multiple myeloma.

Mechanism	Key molecules/pathways	Evidence in MM	Representative evidence	Classification
CD38^+^ Treg depletion	CD38	Strong (*in vitro* + clinical)	Anti-CD38 (Isatuximab) depletes Tregs and enhances CD8^+^/NK cytotoxicity	MM-validated (16)
IFN-I–driven Treg expansion	IFNAR1 signaling	Strong (animal + mechanistic)	IFN-I promotes Treg expansion; IFNAR1 blockade reduces MM progression	MM-validated (17, 18)
APRIL–TACI axis	APRIL, TACI	Strong (MM-specific)	APRIL enhances Treg proliferation and immunosuppression in MM	MM-validated (33)
TGF-β–cGAS-STING suppression	TGF-β1	Moderate–strong (MM mechanistic)	TGF-β suppresses cGAS-STING, downregulates MHC-I	MM-validated (19)
CD39/CD73–adenosine axis	CD39, CD73, A2A receptor	Moderate (MM + tumor evidence)	Adenosine suppresses Teffs and enhances Tregs; partially shown in MM	Partially validated (27–31)
IL-10-mediated suppression	IL-10/STAT3	Moderate (MM + general immunology)	IL-10 inhibits CD8^+^ T/NK cells and maintains Tregs	Partially validated (11, 25, 26)
CTLA-4-mediated inhibition	CTLA-4/CD80/CD86	Limited MM-specific	Well-established in Treg biology; limited MM-specific data	Inferred (13, 14)
Granzyme/perforin cytotoxicity	Granzyme B	Limited MM-specific	Demonstrated in Tregs generally, not MM-specific	Inferred (20)
Treg-mediated angiogenesis	VEGF, CCL28	Weak MM-specific	Mainly extrapolated from solid tumors	Inferred (66–69)

### 2.1 Key molecules mediated by tregs in MM pathogenesis

In the immune microenvironment of MM, certain cytokines and surface molecules regulate the quantity and activity of Tregs, contributing to the immunosuppressive microenvironment in MM and thereby promoting the initiation and progression of the disease. The transcription factor Foxp3, as a specific surface marker of Tregs, is considered a critical regulator of Treg development, function, and stability. It plays a key role in maintaining immune tolerance and immune homeostasis, while also being involved in the occurrence and progression of various diseases. Studies have shown that the continuous expression of the transcription factor Foxp3 determines the immunosuppressive function of Tregs: Fontenot JD and colleagues found that mice with a knockout of the *Foxp3* allele typically die from aggressive lymphoproliferative disease around 4 weeks of age (12). CTLA-4, a pivotal immune checkpoint molecule expressed on Tregs, regulates Teffs activation through dual mechanisms. Studies have demonstrated that CTLA-4 competitively binds to the co-stimulatory molecules CD80/CD86 (B7 family) on dendritic cells (DCs) with higher ligand-binding affinity than CD28 on Teffs. This competitive interaction not only disrupts CD28-mediated co-stimulatory signaling but also actively reduces B7 molecule availability on DCs via trans-endocytosis. Consequently, CTLA-4 exerts dual suppression of both activation and proliferation pathways in Teffs (13, 14). Moreover, activated regulatory T cells can eliminate target cells through perforin-dependent cytotoxicity mediated by granzyme expression, while simultaneously inducing exhaustion of Teffs via direct cell-to-cell contact (15). In the immune microenvironment of MM, CD38 is a key inhibitory molecule highly expressed on the surface of Tregs. Feng et al. conducted an *in vitro* co-culture experiment and found that Tregs exhibit higher levels of CD38 expression compared to conventional T cells (Tcons). Moreover, the proportion of the CD38^+^ Tregs subset is elevated in MM patients compared to healthy individuals. Further research demonstrated that treatment of peripheral blood mononuclear cells (PBMCs) with the anti-CD38 monoclonal antibody Isatuximab (Isa) induces Tregs apoptosis while inhibiting their proliferation and migration. Additionally, Isa significantly suppresses tumor- and bone marrow stromal cell-induced Tregs generation and enhances the cytolytic activity of CD8^+^ T cells and NK cells against MM cells (16).

Animal studies have shown that in the bone marrow of mice injected with myeloma cells, MM cells drive the expansion and activation of Tregs through the secretion of type I interferons (IFN- I). Selective depletion of Tregs in myeloma mice leads to disease remission. Blocking the IFNα and β receptor 1 (IFNAR1) on Tregs with monoclonal antibodies significantly reduces the immunosuppressive function of myeloma-associated Tregs and inhibits myeloma progression (17). The findings of Eskandari et al. are consistent with the above results. Additionally, their study further confirms that IFN-I can enhance the differentiation of naïve CD4+ T cells into Foxp3+ Tregs, a process that relies on ancillary cytokine signals including IL-2 (18). *In vitro* co-culture experiments of Tregs and MM cells from MM patients revealed that Tregs can induce the production of TGF-β1. Subsequent verification suggested that TGF-β1 may inhibit the cyclic GMP-AMP synthase-stimulator of interferon genes (cGAS-STING) pathway, leading to the loss of major histocompatibility complex class I (MHC-I) expression and the upregulation of PD-L1 in MM cells, thereby facilitating immune escape of the tumor cells. Neutralizing TGF-β1 or activating the cGAS-STING pathway restores the expression of MHC-I and PD-L1, effectively counteracting the pro-tumor effects of Tregs on MM cells *in vivo* (19). Other studies have demonstrated that TGF-β1 can directly inhibit the cytotoxic activity of Teffs and NK cells, thereby attenuating the direct immune-mediated killing of tumor cells (20, 21). Additionally, TGF-β1 upregulates the expression of the transcription factor FoxP3 and the anti-apoptotic protein B-cell lymphoma 2 (Bcl-2) (22), which indirectly protects Tregs from apoptosis during their development and promotes the conversion of peripheral blood CD4+ naïve T cells into CD4+CD25+Foxp3+ Tregs, thereby facilitating Tregs generation (23). IL-2, secreted by CD4+ T cells (including naïve T cells, memory T cells, and Th1 cells) upon antigenic stimulation, exerts its biological functions by binding to the IL-2 receptor. The IL-2 receptor complex consists of three subunits: CD25 (α chain), CD122 (β chain), and CD132 (γ chain). The trimeric IL-2 receptor (IL-2R) exhibits significantly higher affinity for IL-2 than the monomeric or dimeric IL-2R. Notably, Tregs express the trimeric IL-2R on their surface, whereas other T cells only upregulate CD25, and NK cells predominantly express the CD122-CD132 dimer. Consequently, Tregs exhibit a significantly higher affinity for IL-2 compared to other T cells or NK cells (24), allowing them to preferentially utilize IL-2 for survival and immunosuppressive functions. In the tumor immune microenvironment of MM, IL-10 is another key immunoregulatory molecule, primarily secreted by immunosuppressive cells such as Tregs, TAMs, and MDSCs. It regulates the immune microenvironment through two main mechanisms: on one hand, by binding to the IL-10 receptor (IL-10R), it inhibits the proliferation and activation of CD8^+^ T cells and NK cells, while also impairing the antigen-presenting function of DCs (11); on the other hand, by activating the STAT3 signaling pathway, it not only maintains the immunosuppressive phenotype of Tregs (25) but also enhances the tryptophan metabolism suppressive activity mediated by arginase 1 (ARG1) and inducible nitric oxide synthase (iNOS) in MDSCs (26), thereby further reinforcing the immunosuppressive microenvironment.

### 2.2 Treg-associated signaling pathways in the pathogenesis and progression of MM

Tregs mediate the immunosuppressive microenvironment in MM by modulating multiple signaling pathways, with the CD39/CD73-adenosine axis playing a central role. Studies have demonstrated that CD39 (ENTPD1) and CD73 (NT5E), co-expressed as functional ectonucleotidases on the surface of Tregs, are significantly enriched in the bone marrow microenvironment of MM patients and exhibit robust enzymatic activity. CD39 initiates the hydrolysis of extracellular ATP to AMP, which is subsequently catalyzed by CD73 into the immunosuppressive metabolite ​adenosine. Adenosine binds to its cognate receptors (e.g., A2A receptor), activating the ​cAMP-PKA signaling cascade. This pathway suppresses the proliferation and cytokine production of Teffs while concurrently upregulating Foxp3 and IL-10 expression to amplify Treg expansion and functional potency, thereby establishing a self-reinforcing positive feedback loop that perpetuates immune evasion (27–30). Notably, MM cells exacerbate adenosine-driven immunosuppression by secreting cytokines such as IL-6 and TGF-β, which directly induce the upregulation of CD39/CD73 on Tregs (31). Flow cytometry analysis of human cancer cell lines directly demonstrated the expression of CD39 on the surface of various tumor cells. Treatment with a CD39 inhibitor or blocking antibody effectively alleviated the tumor-induced suppression of CD4^+^ and CD8^+^ T cell proliferation and significantly enhanced the cytotoxicity mediated by CTLs and NK cells (32). Tai et al. investigated the immunosuppressive role of a proliferation-inducing ligand (APRIL) signaling through the transmembrane activator and CAML interactor (TACI) receptor in the immune microenvironment of MM using a mouse model. Their study revealed that, compared to Tcons, Tregs in MM patients exhibit significantly higher TACI expression, along with upregulation of Treg-associated markers such as Foxp3 and CTLA-4. APRIL promotes Treg proliferation and enhances their anti-apoptotic capacity through TACI, while simultaneously upregulating Foxp3, IL-10, TGFβ1, and PD-L1 expression, thereby reinforcing treg-mediated suppression of Tcons. The use of anti-APRIL monoclonal antibodies effectively blocks these immunosuppressive effects. Furthermore, APRIL enhances the generation of MM-induced inducible Tregs (iTregs) and increases the expression of IL-10, TGFβ1, and CD15s, further augmenting their immunosuppressive function. Additionally, OCs secrete APRIL and PD-L1, promoting iTreg generation and suppressing Tcon proliferation, whereas combined blockade of APRIL and PD-1/PD-L1 effectively reverses this immunosuppressive effect (33).

### 2.3 Interactions between tregs and other immune cells in MM

Tregs interact with Teffs, MDSCs, NK cells, DCs, and TAMs through humoral regulation and cell-to-cell contact mechanisms, collaboratively influencing the initiation and progression of MM.

#### 2.3.1 Tregs and Teffs

Tregs induce exhaustion of Teffs and suppress the effector functions of tumor-specific CD8^+^ and CD4^+^ T cells through direct cell-cell interactions or the secretion of anti-inflammatory cytokines, thereby weakening the anti-tumor immune response, promoting tumor cell proliferation, and facilitating disease progression (34). The development of Tregs and Th17 cells is closely interconnected. Upon TCR stimulation, naïve T cells can express Foxp*3* and differentiate into Tregs under the influence of TGF-β. However, when TGF-β is present alongside IL-6 or IL-21, Treg differentiation is suppressed, and T cells are instead directed toward the Th17 lineage. Notably, IL-17 production by naïve T cells is induced only when TGF-β acts in conjunction with IL-6 or IL-21, rather than in isolation (35). A study utilizing real-time quantitative polymerase chain reaction (RQ-PCR) assessed the expression levels of Foxp3 and retinoic acid-related orphan receptor γt (ROR-γt), a Th17 cell-specific transcription factor, in the bone marrow of newly diagnosed MM patients, solitary plasmacytoma group, and healthy controls. The results showed that Foxp3 was highly expressed in 72% of MM patients, with a 5.89-fold increase compared to the healthy control group. However, ROR-γt expression levels did not differ significantly between MM patients and healthy controls (*P* > 0.05) (36). These findings indicate an elevated Treg/Th17 ratio in MM patients, suggesting a shift toward an immunosuppressive state that may contribute to tumor immune evasion. Another study reached a similar conclusion: flow cytometry analysis revealed that the Treg/Th17 ratio in the MM group was significantly higher than that in the healthy control group (P < 0.05).

#### 2.3.2 Tregs and MDSCs

In various solid tumors and hematologic malignancies, elevated levels of MDSCs in peripheral blood are commonly associated with more advanced tumor stages at diagnosis and larger tumor burden, and may also contribute to the development of chemoresistance (37, 38). The development and immunosuppressive function of MDSCs are jointly supported and regulated by myeloma cells and bystander cells through both direct contact and indirect mechanisms (39). In the microenvironment of MM, MDSCs suppress myeloma-specific T cell immune responses by inducing T cell dysfunction and promoting the development of Tregs. They not only facilitate Treg generation but also interfere with the homing of naïve T cells to lymph nodes. Moreover, MDSCs can induce the differentiation of naïve T cells into iTregs through both TGF-β dependent and independent mechanisms. The TGF-β dependent pathway involves membrane receptor interactions via CD40-CD40L, while the TGF-β independent mechanism is mediated by the secretion of IL-10 and IFN-γ (40). Interestingly, while MDSCs promote the growth and dissemination of MM cells, induce Treg differentiation, and suppress T cell immune responses, MM cells themselves can stimulate the differentiation of precursor cells from healthy donors into MDSCs. This phenomenon highlights a bidirectional regulatory interaction between MDSCs and MM cells (41).

#### 2.3.3 Tregs and NK cells

Multiple studies have emphasized the critical role of NK cells in the anti-MM immune response. In a clinical controlled trial, Vela-Ojeda et al. found that MM patients who responded to thalidomide-based therapy exhibited an increase in NK cell numbers and a decrease in Treg levels, which was associated with improved survival. In contrast, patients who did not respond or responded poorly to the therapy showed reduced NK cell counts, elevated Treg levels, and correspondingly decreased survival rates (42). Furthermore, the study by Kim et al. indicated that in patients receiving lenalidomide combined with dexamethasone, a post-treatment decrease in NK cell expression and an increase in Tregs may be important factors associated with improved survival outcomes (43). These findings suggest that Tregs may influence the initiation, progression, and clinical prognosis of MM by suppressing NK cell function. Mechanistically, activated Tregs can inhibit NK cell proliferation and NKG2D-mediated cytotoxicity through an IL-2–dependent pathway or via surface-expressed TGF-β (44, 45). Numerous studies have demonstrated that, compared to healthy controls, the expression levels of natural killer group 2 member D (NKG2D) on the surface of NK cells are significantly reduced in tumor patients (46), and this reduction may be associated with tumor progression (47). Geng et al. demonstrated through experiments that, in both *in vivo* and *in vitro* environments, Tregs can directly suppress the expression of NKG2D on NK cells via a cell contact-dependent mechanism, thereby weakening their cytotoxicity. Additionally, Tregs increase the secretion of TGF-β and IL-10, promoting immune evasion of MM cells (48).

#### 2.3.4 Tregs and DCs

DCs are important antigen-presenting cells in the body. Based on their developmental origin and functional characteristics, they can be classified into conventional dendritic cells (cDCs), plasmacytoid dendritic cells (pDCs), and monocyte-derived dendritic cells (moDCs), which are sometimes referred to as myeloid dendritic cells (49). In various types of cancer, pDCs are considered to be associated with poor prognosis due to their ability to enhance immunosuppression by promoting the expansion and activation of Tregs (50). Leone et al. reported that during the progression from monoclonal gammopathy of undetermined significance (MGUS) to active or symptomatic MM, there is a progressive accumulation of myeloid dendritic cells (CD11c^+^) and pDCs (CD11c^-^ CD123^+^) in the bone marrow (51); this cellular accumulation is closely correlated with an increased tumor burden (51, 52). Tregs can suppress the antigen-presenting function of DCs through multiple mechanisms. For example, Tregs are capable of depleting peptide–MHC class II complexes from the surface of DCs, thereby impairing their ability to activate T cells (53). When Tregs are co-cultured in direct contact with DCs, they can downregulate the expression of the costimulatory molecules CD80 and CD86 on DCs through a CTLA-4–dependent mechanism, while increasing the expression of soluble PD-L1. This interaction facilitates the formation of Treg–DC conjugates and immunological synapses, thereby interfering with the antigen-presenting function of DCs and inhibiting their secretion of pro-inflammatory cytokines such as TNF-α and IL-12, ultimately enhancing the immunosuppressive effect (54, 55).

DCs also exert regulatory effects on Tregs. Studies have shown that mature autologous DCs can induce CD4^+^ T lymphocytes to acquire an effector-like phenotype. These CD4^+^ T cells, activated by mature DCs, not only possess immunoresponsive capabilities but also exhibit immunosuppressive functions. Moreover, even CD4^+^ T cells that are CD25-negative can acquire immunosuppressive properties and express the key Treg-associated transcription factor Foxp3 upon stimulation by mature autologous DCs (56). Other studies have demonstrated that during tumor progression, tumor cells can promote the proliferation of Tregs by inducing immature DCs to stimulate Tregs in a TGF-β–dependent manner (57). Fiore et al. found that myeloma cells promote immunosuppression by inducing DCs to differentiate into a phenotype that favors Tregs rather than Teffs (58).

#### 2.3.5 Tregs and TAMs

TAMs are among the most abundant immune cell populations infiltrating tumors and are generally classified into two functional subtypes: M1 and M2 macrophages. M1 macrophages typically exhibit anti-tumor activity by directly mediating cytotoxic effects or through antibody-dependent cell-mediated cytotoxicity (ADCC) to eliminate tumor cells. In contrast, M2 macrophages possess pro-tumor properties; they suppress T cell–mediated anti-tumor immune responses, promote tumor angiogenesis, and facilitate tumor initiation and metastasis (59). Moreover, through cell-to-cell contact mechanisms, M2 macrophages can protect myeloma cells from chemotherapy-induced apoptosis (60), thereby playing a critical role in tumor drug resistance and accelerating tumor progression. Studies have shown that under the influence of tumor-derived cytokines, TAMs can be polarized toward an immunosuppressive M2 phenotype (61). By comparing bone marrow tissues from patients with MM and benign controls, it was found that TAM infiltration density was significantly increased in MM patients and positively correlated with microvessel density (MVD), vascular endothelial growth factor (VEGF) expression levels, the proportion of Tregs, and IL-10 levels (62). Studies have shown that tumor cells and TAMs can recruit Tregs by secreting the chemokine CCL22 (63), thereby further suppressing T cell–mediated anti-tumor immune responses. In addition, TAMs and tumor cells can cooperatively produce CCL20, which promotes the recruitment and differentiation of CCR6^+^ Tregs (64). TAMs also express CD39 and CD73, which are involved in adenosine production (65). Thus, Tregs and TAMs interact via the CD39/CD73–adenosine pathway, collaboratively suppressing Teffs function, promoting Treg expansion and differentiation, and contributing to tumor immune evasion and progression.

#### 2.3.6 Mechanistic integration of treg-mediated immunosuppression in MM

Although individual immunosuppressive mechanisms of Tregs have been extensively described, these pathways do not function in isolation. Instead, they form a highly coordinated and interconnected regulatory network within the bone marrow microenvironment of MM. Upstream signals derived from myeloma cells and stromal components, including APRIL–TACI interactions, type I interferons, and immunosuppressive cytokines such as TGF-β and IL-6, promote the expansion, activation, and stabilization of Tregs. These activated Tregs subsequently exert immunosuppressive effects through multiple complementary mechanisms. At the cellular level, Tregs suppress antigen-presenting cell function via CTLA-4–mediated downregulation of CD80/CD86 on dendritic cells, thereby impairing T-cell priming. At the metabolic level, the CD39/CD73–adenosine axis generates an immunosuppressive milieu that inhibits effector T-cell and NK-cell activity through A2A receptor signaling. In parallel, Tregs secrete inhibitory cytokines such as IL-10 and TGF-β, which further suppress cytotoxic immune responses and promote T-cell exhaustion. Notably, TGF-β can also directly modulate tumor cells by suppressing the cGAS-STING pathway and reducing MHC class I expression, thereby facilitating immune escape. These mechanisms converge to establish a self-reinforcing immunosuppressive network that supports tumor immune evasion, promotes disease progression, and contributes to resistance to both conventional therapies and emerging immunotherapies such as CAR-T cells and bispecific antibodies. Therefore, a comprehensive understanding of this integrated regulatory network is essential for the development of effective therapeutic strategies targeting Treg-mediated immunosuppression in MM.

#### 2.3.7 The mechanism by which tregs regulate angiogenesis in the tumor microenvironment of MM

The inhibitory role of Tregs in the bone marrow microenvironment of MM directly or indirectly promotes MM angiogenesis. Tregs indirectly promote angiogenesis by suppressing the activity of Th1 Teffs. This effect is primarily manifested by inhibiting the secretion of anti-angiogenic cytokines (such as TNF-α and IFN-γ) and the expression of interferon-induced chemokines (such as CXCL9, CXCL10, and CXCL11), thereby attenuating anti-angiogenic signals and facilitating the formation of new blood vessels (66, 67). CD4^+^/CD25^+^ Tregs can suppress the activation of tumor-sensitized CD4^+^ T cells, which possess IFN-γ–dependent anti-angiogenic activity (68). Studies have shown that tumor hypoxia can upregulate CCL28, promoting the recruitment of Tregs (69). In a mouse ovarian cancer cell line, forced expression of CCL28 led to a large accumulation of Tregs, accompanied by a significant increase in VEGF levels and enhanced angiogenesis. Notably, depletion of CD25^+^ or CCR10^+^ cells effectively eliminated Tregs from the tumor microenvironment and significantly inhibited local VEGF expression and angiogenesis (69).

Although regulatory T cells have been reported to promote angiogenesis in several solid tumors, direct evidence supporting this mechanism in multiple myeloma remains limited. Angiogenesis is a hallmark of MM progression and has been mainly attributed to interactions between malignant plasma cells, stromal cells, and myeloid cells in the bone marrow microenvironment. At present, the contribution of Tregs to angiogenic processes in MM is largely inferred from studies conducted in other tumor types. Further experimental and clinical investigations are required to clarify whether Tregs directly participate in the regulation of angiogenesis in the MM bone marrow niche.

## 3 The relationship between tregs and treatment response in MM

### 3.1 Tregs and IMiDs

IMiDs, exemplified by thalidomide, exert multiple anti-tumor effects through their synergistic actions on MM cells and the bone marrow microenvironment, including direct inhibition of tumor cell proliferation, suppression of tumor angiogenesis, and modulation of immune responses (70). However, studies on the effects of IMiDs treatment on Tregs are still limited, and the existing research findings remain somewhat controversial.

Yun et al. compared changes in CD4^+^CD25^+^ Tregs in MM patients before and after treatment with thalidomide, suggesting that thalidomide may exert anti-MM effects by downregulating the proportion of Tregs, and that the Treg proportion is closely associated with treatment efficacy (71). A similar conclusion was reached by Giannopoulos et al., who found that, compared to healthy controls, the proportion of Tregs in CD4^+^ T lymphocytes in the peripheral blood of MM patients was significantly higher (72). Additionally, other T cell subsets with immunoregulatory properties, such as CD4^+^CD62L^+^ and CD4^+^GITR^+^ cells, were also significantly increased in MM patients compared to healthy controls, and MM patients with a higher proportion of Tregs had significantly shorter overall survival (OS). However, subgroup analysis showed that in MM patients receiving cyclophosphamide, thalidomide, and dexamethasone (CTD) treatment followed by sequential autologous hematopoietic stem cell transplantation (ASCT), there was no significant change in the proportion of Tregs before and after treatment; in contrast, patients treated with melphalan, prednisone, and thalidomide (MPT) showed an increase in Tregs frequency (72). Studies have shown that pomalidomide and lenalidomide inhibit the proliferation of CD25^+^CD4^+^CTLA-4^+^Foxp3^+^ Tregs; in contrast, thalidomide had no significant impact on the proportion of Tregs throughout the treatment (73). Further research demonstrated that lenalidomide and pomalidomide downregulate Foxp3 expression in Tregs but have no direct effect on Treg survival or apoptosis (73). Studies also indicate that, compared to pre-treatment, the proportion and absolute number of CD4^+^ Tregs in peripheral blood increased after lenalidomide combined with dexamethasone induction therapy (74). Similarly, another clinical study found that in MM patients who relapsed after allogeneic hematopoietic stem cell transplantation, treatment with lenalidomide alone or in combination with dexamethasone chemotherapy led to an increase in the proportion of CD4^+^Foxp3^+^ Tregs in peripheral blood (75).

### 3.2 Tregs and PIs

Studies have shown that the enhanced anti-tumor effect of bortezomib following *in vitro* expansion of Tregs is closely associated with the absence of bortezomib resistance in patients (*P* = 0.022) (76). Shi et al. reported that in patients who responded to bortezomib-based treatment regimens, the proportion of Tregs significantly increased, whereas non-responders showed no significant change compared to baseline (77). Although the absolute number of Tregs was not markedly affected, the treatment influenced the proportions of central memory T cells (T_CM_) and effector memory T cells (T_EM_), reversing the state of T cell exhaustion (77). Similarly, other studies demonstrated no significant change in Tregs among MM patients treated with the BD (bortezomib and dexamethasone) regimen (78). Interestingly, it was found that CD4^+^ T cells cultured in the presence of bortezomib tended to differentiate into Tregs, which subsequently suppressed antigen-stimulated Teffs proliferation, IFN-γ production, and CD40L expression (79). This observation provides a theoretical basis for the use of bortezomib in the treatment of graft-versus-host disease (GVHD). Compared with IMiDs, PIs exert a relatively modest impact on immune cells and the immune microenvironment; thus, their combined effects with other agents on Tregs and immune modulation warrant further investigation.

### 3.3 Tregs and mAbs

Currently, the most widely used monoclonal antibodies in the treatment of MM primarily target CD38. Studies have shown that the use of daratumumab significantly reduces the absolute number of Tregs in the peripheral blood of patients. In high-risk MM patients, the deep and durable responses achieved with daratumumab monotherapy are closely associated with a marked decline in the proportion of Tregs (80). Clinical studies have also demonstrated that isatuximab, another anti-CD38 monoclonal antibody, preferentially suppresses immunosuppressive Tregs by selectively depleting CD38^high Tregs, thereby effectively restoring the anti-myeloma immune response (16). MG1131, an investigational immune checkpoint inhibitor targeting TIGIT, has exhibited multiple immunomodulatory effects in clinical trials. Research indicates that MG1131 enhances NK cell-mediated tumor cytotoxicity, inhibits the suppressive function of Tregs, and restores the interferon-γ secretion capacity of peripheral blood mononuclear cells in MM patients. Overall, this agent exerts a stimulatory effect on T and NK cells while negatively regulating Tregs function (81).

### 3.4 Tregs and cytotoxic agents

In the treatment of MM, cyclophosphamide is one of the most commonly used cytotoxic drugs. In a murine MM model, Sharabi et al. demonstrated that low-dose cyclophosphamide selectively and transiently depletes Tregs. The underlying mechanisms include the downregulation of Bcl-xL and CTLA-4 expression in Tregs, as well as the inhibition of IL-2 secretion by effector T cells, thereby limiting Tregs survival. This immunomodulatory effect contributes to the restoration of peripheral Teffs proliferation and function, ultimately resulting in a significant reduction in MM incidence (82). Additional animal studies have shown that a single administration of low-dose cyclophosphamide induces Tregs depletion lasting only a few weeks. In contrast to a single dose, repeated low-dose cyclophosphamide administration leads to improved survival outcomes (83). Compared with low-dose regimens, high-dose cyclophosphamide exhibits broader cytotoxicity, causing profound depletion of various T cell subsets. However, Tregs typically recover more efficiently than other lymphocyte subpopulations, preventing substantial Tregs loss and thereby failing to reduce the incidence of MM (82).

### 3.5 Tregs and HSCT

The widespread application of ASCT has significantly improved survival in patients with MM and is considered the standard treatment for physically fit MM patients, particularly those with high-risk disease (84). Despite effective induction therapy, ASCT alone often fails to prevent disease progression and relapse, making the reconstitution of immune cell function critically important for the success of subsequent immunotherapies. Chung et al. found that increased levels of Tregs were closely associated with a higher risk of disease progression in post-transplant relapsed patients, whereas decreased Tregs levels were significantly correlated with long-term disease control (85). Similarly, Derman et al. reported that early post-transplant Tregs depletion may represent a feasible and safe immunotherapeutic strategy to delay Tregs recovery after ASCT, thereby enhancing anti-tumor immune responses and reducing the probability of MM relapse (86). Moreover, studies have shown that among patients receiving allo-HSCT, a higher frequency of Tregs was associated with a reduced risk of GVHD (76). In MM patients undergoing ASCT, those with a higher Tregs proportion had longer survival compared to those with lower Tregs levels (87). However, these findings are not consistent with those of Lee et al., who analyzed the T cell phenotypes of bone marrow-infiltrating lymphocytes on day 100 post-ASCT and compared them with those from healthy controls. They found no significant correlation between the frequency of Tregs and post-transplant PFS in MM patients (88).

The prognostic role of Tregs following HSCT is particularly complex and appears to be context-dependent. On one hand, elevated Treg levels may suppress anti-tumor immune responses and promote relapse. On the other hand, Tregs play a critical role in maintaining immune tolerance and preventing excessive immune activation, which may be beneficial during immune reconstitution. This dual role suggests that the timing and functional state of Tregs are more relevant than their absolute numbers. Early depletion of Tregs may enhance anti-myeloma immunity, whereas later recovery may be necessary to restore immune homeostasis. This dynamic balance may partly explain the conflicting clinical observations reported in different studies.

### 3.6 Tregs and emerging immunotherapies

Chimeric antigen receptor (CAR) T-cell therapy targeting B-cell maturation antigen (BCMA) has demonstrated remarkable efficacy in relapsed or refractory MM. However, resistance and relapse remain significant clinical challenges. Preclinical and translational studies suggest that immunosuppressive components of the tumor microenvironment, including Tregs, may inhibit CAR-T cell expansion and persistence, thereby limiting therapeutic durability (89). Evidence indicates that early chimeric antigen receptor T-cell exhaustion and increased regulatory T cells (Tregs) are associated with relapse (90). As noted above, First, Tregs express high levels of CD25 (IL-2Rα), conferring a markedly increased affinity for IL-2 (24). In addition, Foxp3 has been shown to directly repress IL-2 transcription (91). Consequently, excessive competition for and consumption of IL-2 by Tregs deprives CAR-T cells of critical growth signals, thereby impairing their expansion, reducing clonal proliferation, and limiting memory formation. Furthermore, Tregs suppress antigen-presenting cell function through CTLA-4–mediated downregulation of CD80 and CD86 on dendritic cells (54), leading to diminished antigen presentation and costimulatory signaling. Although CAR-T cells are partially independent of MHC-restricted antigen presentation, they still rely on an adequate costimulatory and cytokine milieu for optimal activation and function. Tregs secrete immunosuppressive cytokines such as IL-10, TGF-β, which directly inhibit CAR-T cell effector functions, including cytokine production and cytotoxic activity. Tregs generate extracellular adenosine through the CD39/CD73 pathway, which suppresses CAR-T cell function via A2A receptor signaling. This pathway inhibits cytokine production, reduces cytotoxic activity, and limits CAR-T cell expansion and persistence. The presence of Tregs within CAR-T cell products (CAR-Tregs) represents an additional mechanism of suppression, as these cells can inhibit effector CAR-T function in an autocrine manner, reducing cytotoxicity and limiting therapeutic efficacy (91).

Bispecific antibodies (BsAbs) that redirect T cells to malignant plasma cells have emerged as a promising treatment strategy. In the MajesTEC-1 trial, the BCMA×CD3 bispecific antibody teclistamab achieved an overall response rate of 63% in heavily pretreated MM patients, highlighting the potential of T-cell-engaging therapies (92).

CD3 BsAbs rely on endogenous T cells to mediate antitumor activity, making both T-cell abundance and functional fitness critical determinants of therapeutic efficacy. In MM, however, these parameters are profoundly shaped by the expansion of Tregs, which actively suppress effector T-cell responses. Tregs limit the availability and function of CD8^+^ T cells through high-affinity IL-2 consumption, inhibitory checkpoint signaling, and suppression of T-cell priming, thereby reducing the pool of functional effector cells that can be recruited by BsAbs (93, 94). Moreover, effective BsAb responses require sustained T-cell activation, proliferation, and cytotoxic function following CD3 engagement, a process that is highly dependent on IL-2–driven expansion and continuous effector molecule production. In this context, Treg-mediated IL-2 deprivation and immunosuppressive signaling further restrict T-cell proliferative capacity and durability, thereby impairing the depth and persistence of BsAb-induced antitumor responses (94, 95) Finally, CD8^+^ T-cell exhaustion, a hallmark of MM-associated immune dysfunction, is closely linked to the immunosuppressive activity of Tregs. Through the secretion of inhibitory cytokines such as IL-10 and TGF-β, consumption of IL-2, and expression of checkpoint molecules including CTLA-4 and TIGIT, Tregs contribute to both the induction and maintenance of an exhausted T-cell phenotype. This state is characterized by sustained expression of inhibitory receptors such as PD-1, TIGIT, and LAG-3, along with impaired proliferative capacity and diminished effector function, ultimately rendering CD8^+^ T cells less responsive to BsAb-mediated activation and more prone to rapid functional decline, thereby contributing to primary resistance and limited durability of therapeutic responses (93, 96). Consequently, therapeutic strategies aimed at modulating Treg function or reducing Treg-mediated immune suppression may enhance the efficacy of CAR-T cell therapy and bispecific antibodies in MM (97). Further clinical and mechanistic studies are warranted to explore this possibility.

## 4 Treg heterogeneity and functional specialization in MM

### 4.1 Phenotypic heterogeneity of tregs in MM

Tregs in MM are not a homogeneous population but instead comprise functionally and phenotypically distinct subsets with diverse immunosuppressive capacities. Emerging evidence from flow cytometry, single-cell RNA sequencing (scRNA-seq), and high-dimensional cytometry has demonstrated substantial heterogeneity within the Treg compartment, with important implications for disease progression and therapeutic response (98–100).

In addition to transcriptional diversity, Treg subsets in MM can also be distinguished by surface marker expression linked to functional specialization. Among these, CD38 expression defines a highly suppressive Treg population. CD38^+^ Tregs exhibit increased Foxp3 expression, enhanced proliferative capacity, and greater immunosuppressive activity compared to CD38^-^ counterparts (16). This distinction is of particular clinical relevance, as anti-CD38 monoclonal antibodies may preferentially deplete this subset, thereby contributing to the restoration of anti-tumor immune responses (16).

Similarly, CD39 expression identifies a metabolically specialized subset of Tregs. CD39^+^ Tregs generate immunosuppressive adenosine through the CD39/CD73 pathway, leading to inhibition of effector T cells and NK cells (27–30). F. Marsh-Wakefield et al., however, found that CD39− Tregs exhibited a higher frequency in both the bone marrow and peripheral blood of newly diagnosed MM (NDMM) patients compared to MGUS patients. Using unsupervised FlowSOM clustering, they further identified these cells as activated CD39− Tregs and bone marrow-resident CD39− Tregs (101). This apparent discrepancy may reflect the functional heterogeneity of Tregs in MM. While CD39^+^ Tregs mediate immunosuppression via the adenosine pathway, CD39^-^ Tregs may represent activated or tissue-resident subsets that rely on alternative suppressive mechanisms. Therefore, their increased frequency highlights distinct, non-redundant modes of immune regulation rather than reduced suppressive capacity.

Beyond phenotypic markers, Tregs in MM also exhibit functional heterogeneity characterized by differential expression of immune checkpoint molecules. High-dimensional analyses have identified distinct Treg subsets co-expressing TIGIT and PD-1 in the bone marrow of MM patients (101). In multiple tumor settings, TIGIT^+^ Tregs are associated with enhanced suppressive capacity and preferential accumulation within the tumor microenvironment (102). Recently, Wan Y et al. performed single-cell RNA sequencing by comparing bone marrow samples from three healthy individuals and ten MM Consistently, recent single-cell RNA sequencing has further highlighted the involvement of the TIGIT–NECTIN3 axis in shaping immunosuppressive interactions in the MM bone marrow niche (103). In parallel, PD-1^+^ Tregs display an exhaustion-like phenotype typically associated with effector T cells; however, they retain or even augment their immunosuppressive function, which may contribute to sustained immune suppression and resistance to immune checkpoint blockade (104, 105).

Additionally, functional Treg subsets defined by differentiation status may also influence disease progression. Tregs can be subdivided into resting Tregs (CD45RA^+^FoxP3^lo), activated Tregs (CD45RA^-^FoxP3^hi), and non-suppressive T cells (CD45RA^-^FoxP3^lo). A case–control study including 26 newly diagnosed MM patients, 20 MGUS patients, and 18 healthy controls demonstrated that activated Tregs (aTregs) were significantly increased in both peripheral blood and bone marrow of MM patients, while resting Tregs were decreased. These activated Tregs exhibited stronger suppressive capacity, suggesting that expansion of activated Treg subsets may contribute to immune escape and disease progression (106).

### 4.2 Spatial heterogeneity and microenvironmental adaptation

First, the balance between regulatory T cells and pro-inflammatory Th17 cells represents an important immune parameter influencing disease outcome. Wan Y et al. performed single-cell RNA sequencing, revealing an imbalance in the differentiation pattern of Th17-like cells and an immunosuppressive phenotype associated with MM cells (103). In our previous study involving 130 patients with newly diagnosed MM, we demonstrated that an elevated Treg/Th17 ratio in peripheral blood was significantly associated with inferior progression-free survival (PFS). Patients with a Treg/Th17 ratio >1.0 had a median PFS of 13.87 months, compared with 30.67 months in patients with a lower ratio (*P* = 0.006) (107).

Treg heterogeneity in MM is further shaped by spatial distribution. Bone marrow–resident Tregs are significantly enriched compared to those in peripheral blood and exhibit a more activated and immunosuppressive phenotype (11, 25). These cells often express higher levels of functional markers such as TIGIT (108), and PD-1 (101) reflecting adaptation to the tumor microenvironment. In contrast, peripheral blood Tregs may not fully capture the functional landscape of Tregs within the bone marrow niche.

### 4.3 Heterogeneity as a basis for discrepancies in clinical observations

Notably, as discussed above, the reported frequency and prognostic significance of Tregs in MM remain inconsistent across studies (**Table 2**). These discrepancies are largely attributable to differences in disease stage, e.g., MGUS, NDMM, relapsed/refractory MM (RRMM), sample source (bone marrow vs. peripheral blood), and variability in phenotypic definitions used to identify Tregs. An additional contributing factor is the timing of sample collection. Treg dynamics may vary significantly during different treatment phases (e.g., pre-treatment, induction, consolidation, and maintenance), especially in the context of HSCT where immune reconstitution occurs over time. Moreover, bone marrow–resident Tregs are typically more enriched and functionally active than their peripheral counterparts, while evolving marker strategies (e.g., incorporation of Foxp3, CD127^low, CD39, and TIGIT) may capture distinct Treg subsets with different suppressive capacities. Functionally, Treg frequency alone may not accurately reflect immunosuppressive function, which is more closely linked to cytokine production and metabolic activity. Emerging evidence further suggests that distinct Treg subsets (e.g., CD39^+^, TIGIT^+^, or exhaustion-like Tregs) may exhibit differential responses to therapy, underscoring the limitation of evaluating total Treg populations without accounting for functional heterogeneity (101, 109–111).

**Table 2:** Summary of studies evaluating Treg frequency and function in MGUS and MM.

Study	Disease stage	Sample source	Treg definition	Frequency change	Functional characteristics
Prabhala RH (11)	NDMM	PB	CD4+Foxp3+	↓	↓
	MGUS			↓	↓
Beyer M (122)	NDMM	PB/BM	CD4+CD25^high^Foxp3+	↑	NS
	MGUS	PB/BM		↑	NS
Laronne-Bar-On A (123)	MM Mouse model	BM	CD4+CD25^high^Foxp3+	↑	
		PB		↑	NS
Feyler S (124)	NDMM/LDMM/RRMM	PB	CD4+CD25+Foxp3+	↑	NS
			CD3+CD4-CD8-αβTCR+ (DN Treg)	↓	
		BM	CD4+CD25+Foxp3+/CD3+CD4-CD8-αβTCR+ (DN Treg)	NS	
	MGUS	PB	CD4+CD25+Foxp3+	↑	
			CD3+CD4-CD8-αβTCR+ (DN Treg)	↓	
Brimnes MK (125)	NDMM	PB	CD4+Foxp3+	↑	NS
	LDMM			NS	NS
	MGUS			NS	
Gupta R (126)	NDMM	PB	CD4+CD25+CD127^dim^Foxp3+	↓	NS
Muthu Raja KR (127)	NDMM	PB	CD4+CD25^high^Foxp3+	↑	NS
	RRMM			↑	
	MGUS			NS	
	NDMM		CD8+CD25^high^Foxp3+	↑	NS
Giannopoulos K (72)	NDMM	PB	CD4+CD25^high^Foxp3+	↑	
Bryant C (128)	LTS-MM	PB	CD3+CD4+CD25++CD127-	↑	
Raja KR (129)	NDMM	PB	CD8+CD25^high^Foxp3+	↑	
Fu R (130)	NDMM	BM	CD4+CD25+CD127^low^	↑	
Braga WM (131)	NDMM	BM	CD3+CD4+ CD25^high^Foxp3+CTLA4+	↑	
Frassanito MA (132)	NDMM	PB	CD4+ Foxp3+	↑	
	LDMM			↑	
	NDMM		CD8+ Foxp3+	↑	
	LDMM			NS	
Feng P (133)	MM	PB	CD4+CD25+Foxp3+	↓	
Ma Y (134)	NDMM/LDMM/RRMM	PB	γδTCR+Foxp3+CD27+CD25^high^	↑	NS
Feng X (16)	MM	PB	CD4+CD25^high^Foxp3+	↑	
Wang JN (106)	MGUS	PB	CD4+ Foxp3+	aTreg ↑ rTreg NS	
		BM		aTreg ↑ rTreg ↓	
	NDMM	PB		aTreg ↑ rTreg NS	
		BM		aTreg ↑ rTreg ↓	
Kawano Y (17)	Mouse model	PB/BM	CD4+ Foxp3+	↑	
	SMM	BM	CD4+CD25+ Foxp3+/CD4+CD25+ Foxp3+CD127^low/-^	↑	
Lad D (135)	NDMM	PB	CD45RA-CCR7-Ki67high/ CD45RA-CCR7-Ki67low	↑	
	NDMM	PB	CD4+CD25^high^ CD127^low^	↑	NS
	NDMM	BM	CD4+CD25^high^ CD127^low^	NS	
	MGUS	BM	CD4+CD25^high^ CD127^low^		
Alrasheed N (136)	NDMM	BM	CD4+CD25+ Foxp3+/CD4+CD25+	↑	↑
Kulikowska de Nałęcz A (137)	NDMM/RRMM	PB	CD4+CD25+CD127-/CD4+CD25+Foxp3+/CD4+Foxp3+CD127-	↑	
Bae J (121)	NDMM	PB	CD4+CD25+Foxp3+	↑	
		BM		NS	
	RRMM	PB		↑	
		BM		↑	
	SMM	PB		NS	
		BM		NS	
	MGUS	PB		NS	
		BM		NS	
Noonan K (138)	MM	PB	CD4+CD25+Foxp3+	↑	
		BM		↓	
Zhou Q (139)	NDMM	PB	CD3+CD4+CD25+Foxp3+	ER18>Non ER18	

↑ increase; ↓ decrease; NS, not significant difference; PB, peripheral blood; BM, bone marrow; MGUS, monoclonal gammopathy of undetermined significance; NDMM, newly diagnosed multiple myeloma; RRMM, relapsed/refractory multiple myeloma; LDMM, patients with low disease burden/plateau; DN Treg, double negative tregs; LTS-MM, patients with long-term survival; aTreg, resting Treg; rTreg, activated Treg; ER18, occurring within 18months after initial treatment or within 12months after ASCT; non-ER18, overall survival exceeding 18months and posttransplant survival extending beyond 12months.

Similarly, the impact of therapeutic interventions on Tregs remains controversial, particularly for IMiDs and PIs. These inconsistencies likely reflect both methodological differences and context-dependent immune effects. For IMiDs such as lenalidomide, dual and seemingly opposing effects have been reported: on one hand, lenalidomide can downregulate Foxp3 expression and impair Treg suppressive function (112); on the other, it may enhance IL-2 production (113), thereby indirectly promoting Treg expansion under certain conditions. In contrast, the increase in Treg frequencies observed in responders to proteasome inhibitors may represent a broader immune remodeling process, in which enhanced cytotoxic T-cell and NK-cell activity outweighs the immunosuppressive effects of Tregs (114). In addition, Treg dynamics are influenced by treatment timing and immune reconstitution, particularly in the setting of hematopoietic stem cell transplantation.

## 5 Future directions and clinical translation

Recent advances in Treg-targeting therapies have focused on the development of next-generation CD25-directed agents with improved selectivity and safety profiles. Traditional anti-CD25 antibodies are limited by their concurrent depletion of activated effector T cells; therefore, novel agents have been engineered to preferentially target intratumoral Tregs while sparing peripheral effector populations. For instance, RG6292, a non–IL-2-blocking anti-CD25 antibody, has demonstrated the ability to selectively deplete Tregs through Fc-mediated mechanisms while preserving IL-2 signaling in effector T cells. Early-phase clinical studies in patients with advanced malignancies have shown that RG6292 is well tolerated and exhibits pharmacodynamic evidence of Treg depletion, both as monotherapy and in combination with checkpoint blockade (e.g., atezolizumab) (115, 116). In parallel, antibody–drug conjugates (ADCs) targeting CD25, such as PF-08046032, have been developed to enhance selective cytotoxicity toward Tregs within the tumor microenvironment. Preclinical and early translational data indicate that these agents preferentially eliminate CD25^high Tregs while minimizing systemic toxicity, representing a promising strategy to overcome the limitations of earlier CD25-targeting approaches (117).

Beyond CD25, alternative Treg-depleting strategies are also being explored in clinical trials. For example, CCR4-targeting antibodies (e.g., mogamulizumab) have demonstrated the capacity to deplete effector Tregs in both peripheral blood and tumor tissues, although clinical efficacy as monotherapy remains limited, prompting investigation in combination with immune checkpoint inhibitors (118). Additionally, Fc-engineered antibodies targeting CTLA-4 have been designed to enhance ADCC–mediated depletion of intratumoral Tregs, with early clinical studies showing that the degree of Treg depletion correlates with improved immune activation and clinical outcomes (119). Collectively, these emerging strategies highlight a shift from non-selective Treg depletion toward more precise modulation of the intratumoral Treg compartment, which may be particularly relevant for enhancing the efficacy of T cell–redirecting therapies such as CAR-T cells and CD3 bispecific antibodies.

Emerging evidence highlights that selectively targeting regulatory T cells, particularly CAR-Tregs and tumor-resident Treg subsets, represents a promising strategy to enhance antitumor immunity in MM. Recent studies demonstrate that Timosaponin AIII can significantly enhance CAR-T cell efficacy by impairing the suppressive function of CAR-Tregs, thereby preventing relapse and promoting sustained antitumor responses (91). Mechanistically, this approach alleviates Treg-mediated inhibition within the CAR-T cell product and tumor microenvironment, leading to improved expansion, persistence, and cytotoxicity of effector CAR-T cells. In parallel, targeting CCR8, a chemokine receptor preferentially expressed on clonally expanded, highly suppressive tumor-infiltrating Tregs, enables their selective depletion without broadly affecting peripheral immune homeostasis. Preclinical models have shown that CCR8-directed therapies can induce robust antitumor immunity and durable immune memory, suggesting a high degree of specificity and therapeutic potential (120). Furthermore, co-inhibitory pathways such as LAG-3 and its ligand galectin-3 (GAL-3) have been implicated in reinforcing Treg-mediated immunosuppression in MM. Disruption of the LAG-3/GAL-3 axis not only attenuates Treg suppressive activity but also restores effector T-cell function, thereby enhancing overall antitumor immune responses (121). Collectively, these strategies—targeting CAR-Tregs, CCR8^+^ tumor-resident Tregs, and the LAG-3/GAL-3 immunosuppressive pathway—represent complementary and potentially synergistic approaches to overcome Treg-driven resistance and improve the efficacy and durability of T cell–based immunotherapies in MM.
