# Supplementary figures and images for: The mechanisms of regulatory T cells in the immune microenvironment of multiple myeloma and clinical significance
Source: Front Immunol. 2026 Apr 29;17:1830089. doi: 10.3389/fimmu.2026.1830089 (PMC13168072; doi:10.3389/fimmu.2026.1830089)

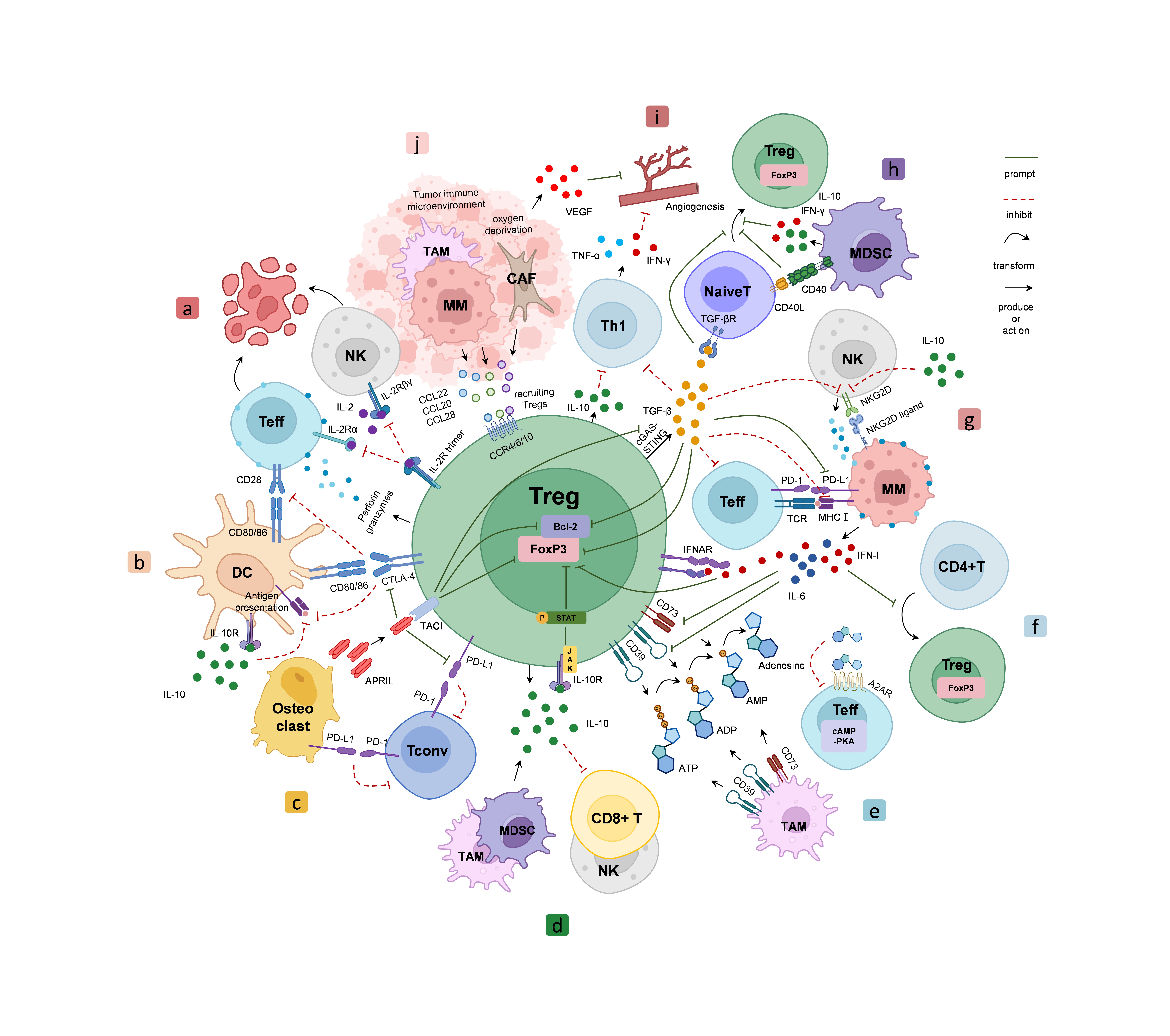

Supplement: Supplementary file 1 [file Image1.jpg]
