# Supplementary material for: The mechanisms of regulatory T cells in the immune microenvironment of multiple myeloma and clinical significance
Source: Front Immunol. 2026 Apr 29;17:1830089. doi: 10.3389/fimmu.2026.1830089 (PMC13168072; doi:10.3389/fimmu.2026.1830089)
Supplement: Supplementary file 2 [file Supplementaryfile1.docx]

Figure S1

The tumor immune microenvironment of MM is characterized by a complex and interdependent network involving various immune cells, cytokines, signaling pathways, surface molecules, and receptors.

a. Tregs exert direct cytotoxicity against effector T cells via perforin and granzyme B. The high-affinity IL-2 receptor trimer on Tregs (CD25/CD122/CD132) competitively sequesters IL-2, depriving NK cells and effector T cells of essential survival signals, ultimately leading to their apoptosis.

b. CTLA-4 expressed on Tregs binds to B7 molecules (CD80/CD86) on DCs, preventing their interaction with CD28 on Teffs, thereby impairing Teff activation and function. Additionally, IL-10 secreted by Tregs inhibits the antigen-presenting capacity of DCs.

c. Osteoclast-derived APRIL binds to its receptor TACI on Tregs, leading to upregulation of CTLA-4, PD-L1, Bcl-2, and FOXP3, thereby enhancing the immunosuppressive phenotype of Tregs and promoting their secretion of TGF-β. In parallel, osteoclasts also exhibit high surface expression of PD-L1, which engages PD-1 on conventional T cells and induces their functional exhaustion.

d. IL-10, mainly produced by Tregs, MDSCs, and TAMs, suppresses CD8⁺ T cells and NK cells, and maintains the suppressive phenotype of Tregs via activation of the STAT3 signaling pathway.

e. CD39 and CD73 expressed on Tregs and TAMs catalyze the hydrolysis of extracellular ATP to adenosine, which binds to A2A receptors on Teffs and inhibits their function through the cAMP-PKA signaling cascade.

f. IFN-I produced by MM cells promotes the differentiation of CD4⁺ T cells into Tregs, enhances the expression of CD39 and CD73 on Tregs, and drives their proliferation and activation.

g. MM cells inhibit Teff function via direct PD-L1/PD-1 interaction. TGF-β upregulates PD-L1 and downregulates MHC-I on MM cells, reducing their immunogenicity. NK cells eliminate MM cells through NKG2D-mediated recognition and cytotoxic granule release, a process attenuated by IL-10 and TGF-β via downregulation of NKG2D on NK cells.

h. MDSCs promote the differentiation of naïve CD4⁺ T cells into Tregs via two mechanisms: a CD40–CD40L-dependent interaction and the production of IL-10 and IFN-γ. TGF-β further reinforces this process by binding to its receptor on naïve T cells.

i. IL-10 indirectly promotes tumor angiogenesis by suppressing Th1 cell-derived TNF-α and IFN-γ production.

j. Under hypoxic conditions, MM cells, TAMs, and cancer-associated fibroblasts (CAFs) upregulate chemokines such as CCL20, CCL22, and CCL28 to recruit Tregs. This recruitment coincides with elevated VEGF levels and contributes to immunosuppression and neovascularization.
